# Personalized application of three different concentrations of iodinated contrast media in coronary computed tomography angiography

**DOI:** 10.1111/jcmm.15196

**Published:** 2020-03-30

**Authors:** Meng Zhang, Panpan Hao, Chenyu Jiang, Guoxiang Hao, Bin Li, Peixin Hu, Qingjie Chen, Yuguo Chen, Aifeng Zhang, Yun Zhang, Yanping Liu

**Affiliations:** ^1^ The Key Laboratory of Cardiovascular Remodeling and Function Research Chinese Ministry of Education Chinese National Health Commission and Chinese Academy of Medical Sciences The State and Shandong Province Joint Key Laboratory of Translational Cardiovascular Medicine Department of Cardiology Qilu Hospital of Shandong University Jinan China; ^2^ Shandong Institute of Innovation Suzhou Institute of Biomedical Engineering and Technology Affiliated with Chinese Academy of Sciences Jinan China; ^3^ Department of Clinical Pharmacy School of Pharmaceutical Sciences of Shandong University Jinan China; ^4^ Jinan Central Hospital Affiliated with Shandong First Medical University and Shandong University Jinan China; ^5^ First Hospital Affiliated with Xinjiang Medical University Urumqi China; ^6^ Department of Nephrology Brigham and Women's Hospital Affiliated with Harvard Medical School Boston Massachusetts; ^7^ Department of Radiology Qilu Hospital of Shandong University Jinan China

**Keywords:** body surface area, contrast media, coronary CT angiography

## Abstract

No study has evaluated the impact of different iodinated contrast media on coronary contrast enhancement, using an injection protocol according to body surface area (BSA). Thus, the present study aimed to examine the usefulness and safety of personalized application of different iodine concentrations of contrast media in coronary computed tomographic (CT) angiography with a 2nd dual‐source CT scanner in eliminating differences in coronary contrast enhancement based on a BSA‐adapted injection protocol of contrast media. A total of 270 enrolled participants were randomly assigned to three groups: ioversol 320, ioversol 350 and iopromide 370 (n = 90 per group). The three groups were administered contrast media at a BSA‐adjusted volume and flow rate with a fixed injection time of 15 seconds, and they subsequently received a 30‐mL saline flush. All patients were scanned with a prospective electrocardiogram‐gated protocol in a craniocaudal direction using a second‐generation 128‐slice dual‐source CT system. The three iodinated contrast media used in coronary CT angiography exhibited similar diagnostic quality and safety. No significant differences were found in the contrast enhancement degrees, image quality scores, radiation doses and incidences of adverse effects among the three groups. The three contrast media used in coronary CT angiography with 320, 350 and 370 mg/mL iodine, respectively, have comparable diagnostic quality and safety. However, more large‐scale, multinational, multi‐centre and prospective trials are warranted.

## 1 INTRODUCTION

Non‐invasive computed tomography (CT) angiography, especially coronary CT angiography, has attracted increasing attention and has been widely used in the diagnosis of cardiovascular diseases. These CT devices include a 64‐row spiral CT scanner, post‐64‐row CT scanner and dual‐source CT scanner.^1^ With high rack speed and time resolution, wide detector coverage, and little restriction of heart rate, dual‐source CT can facilitate prospective electrocardiogram‐triggering scan mode and automatic tube voltage regulation technology to reduce radiation dose.^2^

Iodinated contrast agents are widely applied in CT enhancement. The iodine concentration is associated with the osmotic pressure and viscosity of contrast agents, and the amount of contrast agents might be an independent risk factor for contrast‐induced nephropathy.^3^ Therefore, the selection of optimal contrast agents, optimal iodine concentrations and minimum amount of contrast agents is critical for medical safety.

Previous studies demonstrated that at least 325‐Hounsfield unit (HU) enhancement levels in the coronary arteries were necessary for establishing an accurate diagnosis. Low enhancement levels yielded poor contrast between the arteries and their surrounding tissues, which was unfavourable for diagnostic accuracy. High enhancement levels produced steak artefacts and obscured small calcified plaques.^4^ Arterial attenuation is determined by injection parameters, scanning parameters and patient characteristics. The injection parameters include contrast agent concentration, contrast agent viscosity, contrast agent volume, injection temperature and velocity, iodine dosage and delivery rate, saline flush, needle type and so on. The scanning parameters include tube voltage, scanning duration, scanning delay, tube current and so on. The patient factors include heart rate, blood volume, height, bodyweight, body surface area (BSA), respiratory rhythm, heart function, renal function and other various pathologic conditions.^5^

To minimize the effects of bodyweight and/or body mass index (BMI) on image noise, protocols of contrast agent injection according to bodyweight and/or body mass index (BMI) have been suggested recently.^6^, ^7^ However, coronary contrast might be different because of the variation in blood volume.^8^, ^9^, ^10^ As BSA is positively correlated with blood volume, it has been considered as the most promising factor to adjust contrast bolus.^8^ Moreover, no study has reported the effects of different iodinated contrast media on coronary contrast enhancement, using a standardized injection protocol based on BSA or bodyweight.

Therefore, the present study aimed to evaluate the usefulness and safety of the personalized application of different iodine concentrations of contrast media in coronary CT angiography with the second‐generation dual‐source CT scanner in eliminating differences in coronary contrast enhancement based on the BSA‐adapted injection protocol.

## 2 MATERIALS AND METHODS

### 2.1 Ethics statement

The study protocol was submitted to the ethics committee of Qilu Hospital of Shandong University and was approved before inception. Written informed consent was provided by all participants.

### 2.2 Participants

We enrolled 270 consecutive participants suspected of having coronary artery disease between February 2016 and October 2016 at Shandong University Qilu Hospital and Shandong Provincial Medical Imaging Institute, Jinan, China. We excluded the following patients: (a) patients with previous allergic reaction to iodinated contrast media; (b) patients with renal or hepatic dysfunction; (c) patients with heart failure with NYHA IV or left ventricular ejection fraction ≤30%; (d) patients who had undergone pacemaker implanted surgery, coronary stenting or coronary artery bypass grafting previously; (e) patients with other cardiovascular diseases (eg cardiomyopathy, congenital heart disease and valvular disease); (f) patients with pulmonary hypertension; and (g) patients with frequent episodes of arrhythmia or heart rate >90 times/min after oral administration of metoprolol.

For each patient, age, sex, weight, height, resting heart rate, blood pressure and history were recorded in detail, and BMI and BSA were calculated and recorded.

### 2.3 Injection and scanning protocol

A total of 270 enrolled patients were randomly assigned to three groups (n = 90 per group) based on the iodinated contrast medium used. Ioversol 320 (320 mg/mL iodine, Hengrui), ioversol 350 (350 mg/mL iodine, Hengrui) and iopromide 370 (370 mg/mL iodine, Bayer) were, respectively, administered in the three groups for 15 seconds with a 30‐mL saline flush. The volumes and injection rates of contrast media were adjusted according to the BSA,^11^ with a volume of 36.5, 33.3 and 31.5 mL per BSA and a flow rate of 2.4, 2.2 and 2.1 mL/s per BSA (Table 1). BSA was calculated using the following formula: BSA (m^2^) = square root of (height (cm) × weight (kg)/3600). Iodine load was calculated as the concentration multiplied by total volume of contrast media, and iodine delivery rate was calculated as the concentration multiplied by injection rate of contrast media. Contrast agent injection was timed using bolus tracking in the descending aorta, and CT examinations were performed after 10 seconds over 100 HU.

**TABLE 1:** Contrast agent protocols used in the three groups

Iodine concentration (mg/mL)	Volume (mL)	Flow rate (mL/s)	Fixed injection time (s)
370 (Iopromide)	31.5BSA	2.1BSA	15
350 (Ioversol)	33.3BSA	2.2BSA	15
320 (Ioversol)	36.5BSA	2.4BSA	15

Abbreviation: BSA, body surface area.

A second‐generation 128‐slice dual‐source CT scanner was used to conduct all examinations (SOMATOM Definition Flash from Siemens, Forchheim, Germany) as previously described.^12^ The CT examination was performed using a prospective electrocardiogram‐gated protocol in a craniocaudal direction, and the scanning parameters were identified according to the heart rate and BMI of each patient.^12^ The CT scanner could record the CT volume dose index (CTDI_vol_) and dose‐length product (DLP). DLP (mGy∙cm), as an indicator of patient dose from CT tube radiation output/exposure, was measured as CTDI_vol_ (mGy) multiplied by scan length (cm). The effective radiation dose (ED) was calculated by multiplying DLP by 0.014 mSv/(mGy∙cm).

### 2.4 Image quality assessment

The images were reconstructed using the sinogram‐affirmed iterative reconstruction algorithm (SAFIRE, Siemens). The adjustable increment of strength 1 was used for the adaptation of the noise model. Transverse images were reconstructed at 0.75‐mm slice thickness with a 0.5‐mm increment and a matrix size of 512 × 512. All images were anonymized and transferred to a picture archiving and communicating system (syngo MMWP VE 36A, Siemens). By using axial data, multiple intensity projection, multiple planar reformation, curved planar reformation and volume‐rendering images of the coronary arteries (the thresholds for image segmentation ranging from −9 to 645 HU) were reconstructed for further analyses.

Image quality was assessed by two specialists blindly and independently, and any inter‐observer disagreement was resolved through discussion after re‐reading the images. Images with severe artefacts and no diagnostic value were scored as 1; poor images with substantial artefacts or pixel noises and moderate impairment of diagnostic value were scored as 2; fair images with clearly visible artefacts or pixel noises but no impairment of diagnostic value were scored as 3; good images with scarcely visible artefacts or pixel noises were scored as 4; and excellent images without artefacts or pixel noises were scored as 5.^13^ Image quality of the ascending aorta, right coronary artery (RCA), left main coronary artery (LM), left anterior descending artery (LAD) and left circumflex (LCX) was evaluated at the same three anatomic levels that were used for quantitative analyses of the arterial enhancement. Images with a score of 3 or greater were considered to be of diagnostic value.

### 2.5 Statistical analysis

The SPSS v19.0 software was used for the statistical analyses. Inter‐group differences were analysed by Welch's test, the Mann‐Whitney U test or the independent sample *t* test. Two‐tail *P* < .05 was defined as statistically significant.

## 3 RESULTS

### 3.1 Baseline characteristics of the participants

No significant differences in sex, age, bodyweight, height, BMI, BSA, heart rate and blood pressure were noted among the 3 groups (Table 2).

**TABLE 2:** Basic characteristics of the participants

	Iopromide 370	Ioversol 350	Ioversol 320	*P*
n (Male/Female)	90 (48/42)	90 (45/45)	90 (52/38)	>.05
Mean age (years)	52.79	51.77	53.74	>.05
Mean weight (kg)	70.92	70.71	70.07	>.05
Mean height (cm)	166.43	167.21	165.04	>.05
Mean BMI (kg/m^2^)	25.50	25.29	24.71	>.05
Mean BSA (m^2^)	1.81	1.81	1.79	>.05
Mean heart rate (bpm)	66.68	67.62	69.92	>.05
Mean SBP (mm Hg)	136.03	136.14	136.28	>.05
Mean DBP (mm Hg)	84.17	83.69	84.07	>.05

Abbreviations: BMI, body mass index; BSA, body surface area; DBP, diastolic blood pressure; SBP, systolic blood pressure.

### 3.2 Image quality assessment

**FIGURE 1:**
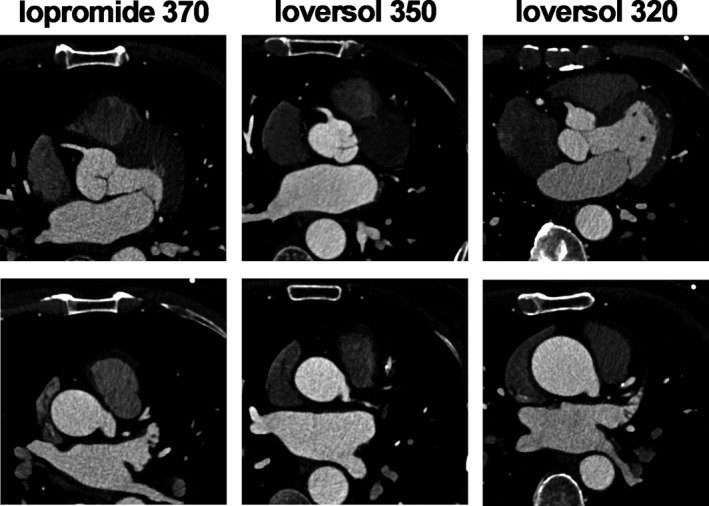
Coronary sinus imaging in the three groups

**FIGURE 2:**
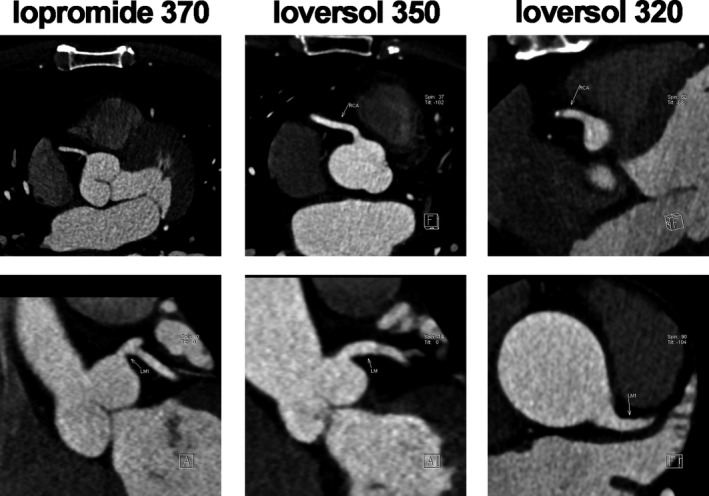
Proximal coronary imaging in the three groups

**FIGURE 3:**
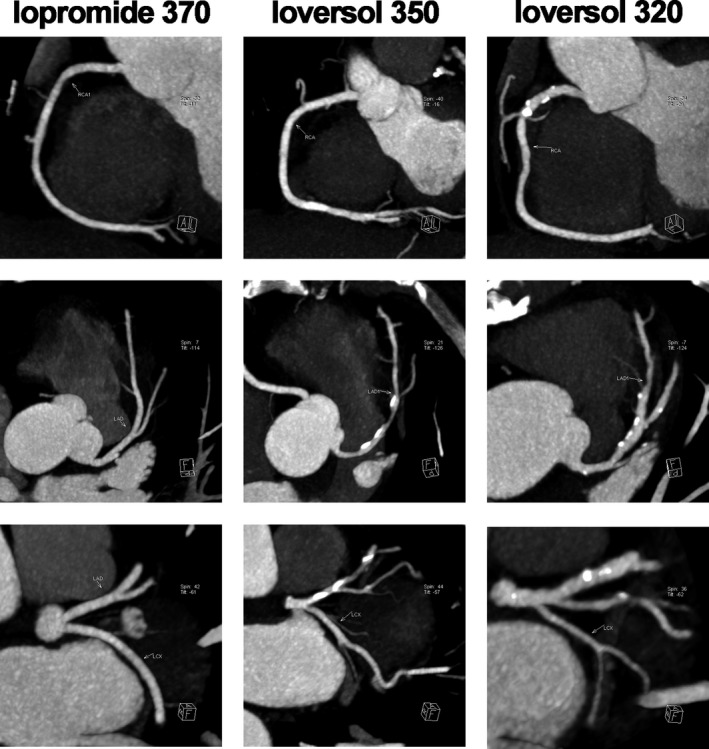
Coronary artery trunk imaging in the three groups

The mean CT values of noise in the iopromide‐370 group, ioversol‐350 group and ioversol‐320 group were 21.14 HU, 21.43 HU and 21.86 HU, respectively; the mean CT values of the ascending aorta in the three groups were 473 HU, 485 HU and 485 HU, respectively; the mean CT values of the RCA in the three groups were 443 HU, 453 HU and 446 HU, respectively; the mean CT values of the LM in the three groups were 476 HU, 466 HU and 472 HU, respectively; the mean CT values of the LAD in the three groups were 457 HU, 455 HU and 451 HU, respectively; and the mean CT values of the LCX in the three groups were 429 HU, 441 HU and 432 HU, respectively. No significant differences were found in the CT values of noise, aorta, RCA, LM, LAD and LCX among the three groups (Table 3). The mean image quality scores in the 3 groups were 4.16, 4.13 and 4.11, respectively. No significant difference was found in the image quality scores among the 3 groups (Table 4; Figures 1, 2, 3).

**TABLE 3:** Coronary artery attenuation

Mean CT (HU)	Iopromide 370	Ioversol 350	Ioversol 320	*P*
Noise	21.14	21.43	21.86	>.05
Ascending aorta	473	485	485	>.05
RCA	443	453	446	>.05
LM	476	466	472	>.05
LAD	457	455	451	>.05
LCX	429	441	432	>.05

Abbreviations: LAD, left anterior descending artery; LCX, left circumflex; LM, left main coronary artery; RCA, right coronary artery.

**TABLE 4:** Image quality scores in the three groups

	Iopromide 370	Ioversol 350	Ioversol 320	*P*
Mean image quality score	4.16	4.13	4.11	>.05

### 3.3 Volumes and flow rates of contrast agents

**FIGURE 4:**
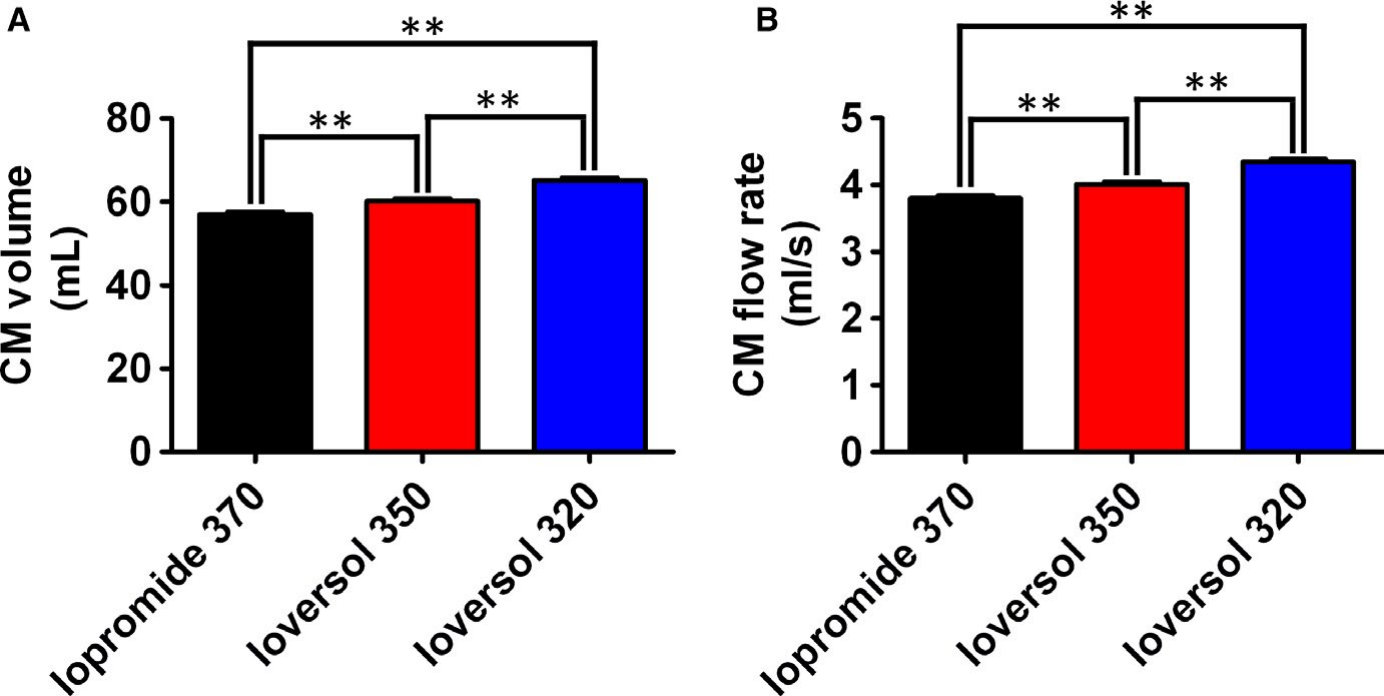
Quantification of the contrast agent volumes and flow rates in the three groups. **P < .01

The mean volumes of iopromide 370, ioversol 350 and ioversol 320 were 56.98 mL, 60.20 mL and 65.21 mL, respectively. There were significant differences between any two groups among the three groups (*P* < .01). The mean flow rates in the three groups were 3.80 mL/s, 4.01 mL/s and 4.35 mL/s, respectively. There were significant differences between any two groups among the three groups (*P* < .01). The use of lower concentrations of contrast media required higher volumes and flow rates, and the use of higher concentrations of contrast media required lower volumes and flow rates (Table 5; Figure 4).

**TABLE 5:** Contrast agent volumes and flow rates in the three groups

	Iopromide 370	Ioversol 350	Ioversol 320	*P*
Mean volume (mL)	56.98	60.20	65.21	<.05
Mean flow rate (mL/s)	3.80	4.01	4.35	<.05

### 3.4 Safety assessment

No significant difference was found in radiation dose among the three groups (*P* > .05, Table 6). All patients had no serious adverse reactions during the CCTA examination, including allergic shock, contrast agent leakage, acute renal impairment, acute myocardial infarction, arrhythmia, vascular complications (bleeding, embolization and so on) and vasovagal reflexes.

**TABLE 6:** Radiation dose assessment in the three groups

	Iopromide 370	Ioversol 350	Ioversol 320	*P*
Mean DLP (mGy*cm)	302.34	292.12	290.08	>.05
Mean ED (mSv)	4.23	4.09	4.06	>.05

Abbreviations: DLP, dose‐length product; ED, effective radiation dose.

## 4 DISCUSSION

The present study evaluated the usefulness and safety of the BSA‐adapted application of three contrast media with different iodine concentrations in coronary CT angiography with the 2nd dual‐source CT scanner. The results demonstrated no significant differences in the degrees of contrast enhancement, image quality scores, radiation dosages and incidences of adverse effects. As mentioned above, the three iodinated contrast media used in coronary CT angiography exhibited similar diagnostic quality and safety.

The impact of iodine concentration on attenuation remains controversial. Administration of different iodinated contrast media exhibited similar vessel attenuation in numerous studies.^14^, ^15^, ^16^, ^17^ Conversely, some studies attributed higher attenuation to higher iodine concentrations.^18^, ^19^, ^20^ A previous study that assessed and compared the diagnostic quality of iodixanol (320 mg/mL iodine) and iomeprol (400 mg/mL iodine) at the same flow rate (5 mL/s) and volume (80 mL) showed that higher iodine concentrations with constant flow rate and volume exhibited more benefit in terms of coronary attenuation. The different iodine delivery rates (cumulative effects from iodine concentration and flow rate) contributed to the attenuation difference between the two groups.^20^ A previous study that compared five different contrast agents with a constant flow rate and volume found comparable findings for all the five contrast agents.^18^ The iodine concentrations were positively correlated with the mean attenuation values. In addition, the iodine delivery rates differed (1.2‐1.6 g/s) with a constant flow rate among the groups.^18^ The coronary attenuation levels can be used for diagnosis when iodine delivery rates were at least 1.4 g/s. The iodine delivery rate is more feasible to be regulated via adjustment of injection rates than via replacement of contrast agents, although it is affected by both iodine concentration and flow rate.^21^

As some studies reported, when the iodine delivery rate and total iodine load remained unchanged, administration of different iodinated contrast media resulted in comparable attenuation levels, image quality and incidences of adverse reactions.^22^, ^23^, ^24^, ^25^ According to these studies, under the situation of identical iodine delivery rate and injection and scanning parameters, the equivalent vessel enhancement patterns were built using variable contrast media at iodine concentrations from 240 to 400 mg/mL.^22^, ^23^, ^24^, ^25^ Our results also showed that the injection rate was not associated with any adverse reaction. As contrast media with low iodine concentrations have low osmolality and viscosity, our findings suggest the application of low iodine concentrations (320 mg/mL and even lower concentration) with high flow rates for personalized contrast protocols.

Cigarroa et al proposed the following formula for the amount of contrast media: bodyweight (kg) × 5 mL/serum creatinine level (SCr; mg/dL), with the maximum amount being no more than 300 mL. The results demonstrated that the incidences of contrast‐induced nephropathy were 21%‐37% above the threshold dose and 0%‐2% under the threshold dose.^26^ Application of contrast agents based on BMI or BSA might be more accurate than that based on bodyweight.^7^ In a previous study, an individually BSA‐adjusted contrast agent protocol resulted in excellent image quality, with a significant reduction in the amount of contrast media.^27^ Our study formulated a more individualized BSA‐adjusted contrast agent protocol with a fixed iodine flow dose per BSA.

It has been proved that saline chaser after contrast agent injection is necessary, as it could help to utilize the contrast agent remnants and avoid the remainder in the tubing and peripheral veins.^21^, ^28^ Forty‐two participants received the same iodine delivery rate of 1.28 g/s and were randomized into two groups according to saline flush or the same volume of contrast agent. Administration of 100 mL of contrast agent followed by a 40‐mL saline flush exhibited coronary attenuation not significantly different from that obtained by administration of 140 mL of contrast agent. Some contrast agent was retained in the peripheral veins before the saline flush, and thus, the procedure was not effective in increasing coronary attenuation.^29^

Iodinated contrast agents include ionic dimers, non‐ionic dimers, ionic monomers and non‐ionic monomers based on their chemical structures and ionization in solution. The sole ionic dimer is ioxaglate, which has an iodine concentration of 320 mg/mL and an osmolality of 600 mOsm/kg H_2_O. Non‐ionic dimer iodixanol has an iodine concentration at 320 mg/mL and the same osmolality as plasma, but its high viscosity (26 cPs at 37°C) limits its clinical application. The osmolality of ionic monomers is about twice as that of non‐ionic monomers at the same iodine concentration. Thus, non‐ionic monomers, especially ioversol and iopromide, are widely used in clinical practice owing to their lower osmolality and their association with fewer chemotoxic side effects than the ionic monomers.^30^, ^31^

## 5 CONCLUSIONS

The three contrast agents used in coronary CT angiography with iodine concentrations of 320 mg/mL, 350 mg/mL and 370 mg/mL, respectively, have similar diagnostic quality and safety. However, more large‐scale, multinational, multi‐centre and prospective trials are warranted.

## CONFLICT OF INTEREST

The authors confirm that there are no conflicts of interest.

## AUTHOR CONTRIBUTIONS

Zhang M, Hao P, Liu Y, Jiang C, Hao G, Li B, Hu P, Chen Q and Chen Y performed the trial; Zhang M, Hao P, Zhang A and Liu Y analysed the data and did the statistical analysis; Liu Y, Zhang Y and Hao P designed the study protocol and wrote the manuscript.

## Data Availability

The data that support the findings of this study are available from the corresponding author upon reasonable request.
